# Impact, impact, impact

**DOI:** 10.1093/jhps/hny035

**Published:** 2018-10-30

**Affiliations:** Journal of Hip Preservation Surgery

Oh dear, I have lost count of the number of times someone in publishing has complained. Not about me, I should hasten to add, but at how journals are compared. Invariably the complaint is about the impact factor, the IF, that journal metric we love to hate, that metric that simply will not go away.

For some reason, we love to be published in journals with an IF that is sky high, as by implication it means that our paper was more than marvellous. That is not the case, of course, as even in truly high impact journals, up to 40% of papers may never be cited. The IF is a measure designed for a journal, not the papers it contains. Even its founder, Garfield [1], highlighted the skewed nature of the metric, where 80% of a journal’s citations might be created by 20% of its papers.

The IF was originally introduced in 1955 [2] as a way of libraries choosing which journals to purchase, but matters appeared thereafter to get rather out of hand. In an attempt to right what many see as an increasing wrong, the 2012 San Francisco Declaration on Research Assessment stated as its first recommendation that we should ‘not use journal-based metrics, such as Journal Impact Factors, as a surrogate measure of the quality of individual research articles, to assess an individual scientist’s contributions, or in hiring, promotion, or funding decisions’. It went on to encourage publishers to ‘Greatly reduce emphasis on the journal impact factor as a promotional tool …’ [3]. To date the declaration has been signed by 503 organizations and 12 466 individuals [4]. I suspect there are plenty more to come although this challenge to the established order was not supported 2 years later, with the description of the author impact factor [5], to me a worrisome development.

Years ago, I applied for an overseas academic post. I did not get it, of course, but my curriculum vitae was returned splattered with pencil scribblings. An assessor had been truly busy and had laboriously gone through each of my more than 200 publications and worked out the impact factor for every journal in which my research had appeared. I was unceremoniously kicked into touch. Yet there are signs that the worm is turning. Take the journal *Nature* as an example. Who would not sacrifice a considerable amount to be published in that? But although journals such as *Nature* are publishing an increasing number of high-citation papers, they are not keeping up with the market. Between 1997 and 2012, for example, the number of papers submitted to *Nature* increased by 43%. During the same period, the number of papers published worldwide increased by 86% [6], so clearly researchers are seeking alternatives.

So, I become restless when folk ask me if it is time for this journal, *JHPS*, to seek an impact factor, as the moment we do so, our journal will be stood alongside so many others. We will be compared to them purely on numerical grounds. To me, and to plenty of others [7], the most important criterion for a journal is the quality of its papers. I think you will agree that the quality we find on the digital pages of *JHPS* is astonishingly impressive. Our Editorial Board, too, is well-known, hard-working, eminent and actively participates in the journal’s decisions and future shape. And my fellow Editors and Editorial Correspondents are, to put it mildly, utterly brilliant.

Just look at our last issue, in which I found it almost impossible to select papers to consider. Each was first rate. There was the excellent review article by Michael Wyatt and Martin Beck on the management of the painful borderline dysplastic hip [8], an area with which I have long struggled. And I was very impressed at the simultaneous acetabular labrum and ligamentum teres reconstructions that were reported by White *et al.* [9]. True arthroscopic talent. I have done both but never at the same time. A remarkable case to report.

And if our last issue was not enough, try this one, issue 5.3. In recent times we have been receiving a huge number of papers, so thanks to all who have taken the plunge and realised that *JHPS* is difficult to beat. Each submission seems almost better than the last. Again, being unable to pick a favourite as I simply cannot decide, I was especially interested in the paper by Davies *et al.*, who help identify factors increasing the risk of failure after hip arthroscopy [10]. What an excellent contribution. It is far better to know such things before you start. I was pleased also to read the update on osteonecrosis, and the role of cell therapies and hip arthroscopy in its management, from Papavasiliou *et al.* As the authors state, it is only rational to consider the use of cell-based treatments to potentially regenerate lost or damaged bone [11].

So, as ever, please enjoy this issue of *JHPS*. It is published for you, the hip preservation practitioner, and is filled from cover to cover with brilliance. I commend this issue to you in its entirety.

My very best wishes to you all.
